# Pentraxin-3 in thyroid nodules: a systemic marker of pathology and a local mediator of inflammation and carcinoma

**DOI:** 10.3389/fendo.2026.1847072

**Published:** 2026-07-17

**Authors:** Miriam Cieri, Fabio Grizzi, Barbara Bottazzi, Giorgia Amy Rodda, Paola Petrillo, Roberto Leone, Fabio Pasqualini, Emanuela Morenghi, Walter Zuliani, Silvia Uccella, Damiano Chiari

**Affiliations:** 1 Department of Biomedical Sciences, Humanitas University, Pieve Emanuele, MI, Italy; 2 Department of Pathology, IRCCS Humanitas Research Hospital, Rozzano, MI, Italy; 3 Department of Immunology and Inflammation, IRCCS Humanitas Research Hospital, Rozzano, MI, Italy; 4 Laboratory Analysis, Humanitas Mater Domini Clinical Institute, Castellanza, VA, Italy; 5 Biostatistics Unit, IRCCS Humanitas Research Hospital, Rozzano, MI, Italy; 6 General Surgery Department, Humanitas Mater Domini Clinical Institute, Castellanza, VA, Italy

**Keywords:** cancer, immunity, inflammation, pentraxin 3, thyroid

## Abstract

**Background:**

Thyroid nodules are extremely common, yet the exact physio-pathological mechanisms underlying their benign or malignant development remain poorly understood. Pentraxin-3 (PTX3) is an innate immune inflammatory mediator involved in inflammation, tissue remodeling, and oncogenesis, processes often coexistent in thyroid pathology. This study evaluated plasma and tissue PTX3 in patients undergoing thyroidectomy for benign or malignant nodules.

**Methods:**

A total of 52 patients were enrolled. Plasma PTX3 levels were assessed by ELISA before surgery and again 45 days afterward. PTX3 expression, along with the abundance of two immune cell populations (e.g. macrophages and mast cells) was evaluated by immunohistochemistry using a tissue microarray.

**Results:**

Preoperative plasma PTX3 levels were significantly elevated (4.58 ng/mL, p<0.05) compared with the normal reference range and declined significantly after surgery (3.58 ng/mL, p<0.05). No significant differences in plasma PTX3 were observed between benign and malignant conditions. In tissue analysis, PTX3 expression was significantly elevated in malignant tissues relative to healthy thyroid counterparts, in which it was entirely absent; however, its overall expression levels remained low to moderate across most malignant samples. CD68 and tryptase showed variable patterns, with a higher abundance of macrophages and mast cells in carcinomas and a significant association between mast cell presence and thyroid cancer (p = 0.0025).

**Conclusions:**

Our findings suggest that increased PTX3 levels may indicate an active inflammatory response or tissue stress linked to thyroid nodular disease. Nevertheless, its specificity for distinguishing benign from malignant lesions has yet to be determined. Its strong local expression in thyroiditis and consistently low expression in most carcinomas further support the concept that the long pentraxin PTX3 plays an important role in innate immune activation and inflammation-driven thyroid tissue remodeling. Additional studies are needed to better define its potential prognostic value in thyroid carcinoma.

## 1 Introduction

Thyroid nodules are extremely common (1). Palpable nodules are present in 5% of the population and they are discovered incidentally during ultrasound in 70% of the population (2). While almost 90% of thyroid nodules are benign, thyroid carcinoma is found in 4-15% of the cases (3). At present, many progresses are reached in early diagnosis and management of thyroid nodules and carcinomas but very little is known about the physio-pathological mechanisms behind their development (4, 5). Pentraxin 3 (PTX3) is a prominent member of the long-chain pentraxin subfamily within the broader pentraxin family, which also includes well-known short-chain members such as C-reactive protein (CRP) and serum amyloid P (SAP) (6, 7). As a novel acute-phase inflammatory mediator, PTX3 plays a critical role in the innate immune response to infectious diseases. In addition to its role in inflammation, PTX3 is involved in a range of biological processes, including immune regulation, tissue remodeling, and oncogenesis (8–11). These processes often coexist in the thyroid, but the role of PTX3 in thyroid disease is still unknown (12, 13). PTX3 is a fluid-phase pattern recognition molecule that acts as a key component of the humoral branch of innate immunity (6–8). Beside playing a role in antimicrobial resistance against selected microorganisms (11, 14, 15), PTX3 is produced by several cell types during tissue damage and stress. It is also continuously produced and stored in lactoferrin granules of neutrophils (16) in a ready to-use-form released in response to microorganisms. PTX3 expression is induced by various inflammatory stimuli, including cytokines, Toll-like receptor (TLR) agonists, microbial components such as lipopolysaccharide (LPS), and whole microorganisms. These signals stimulate PTX3 production in a wide range of cell types, including dendritic cells, monocytes, macrophages, endothelial cells, alveolar epithelial cells, adipocytes, and fibroblasts (6). Furthermore, a PTX3 deficiency has been associated with an increased inflammatory response and tissue damage (8). Bonavita and colleagues found that PTX3 acts as an extrinsic oncosuppressor in mice and humans regulating complement-dependent tumor-promoting inflammation (9). In thyroid nodules inflammation, tissue remodeling and oncogenesis are often coexistent (13). Currently there are limited evidence of PTX3 role in thyroid disease. Wang et al. have demonstrated a TSH-induced expression of PTX3 in fibrocytes and orbital fibroblast in Graves’ ophthalmopathy, identifying a possible connection between the thyroid axis and tissue remodeling (12). A recent study identified a four genes signature including PTX3, 3’-phosphoadenosine 5’-phosphosulfate synthase 2 (PAPSS2), procollagen C-endopeptidase enhancer 2 (PCOLCE2) and transforming growth factor beta receptor 3 (TGFBR3) for papillary carcinoma as marker for risk stratification and survival prediction. PTX3 expression was significantly lower in patients surviving with tumors than in tumor free patients, revealing a potential correlation between PTX3 and tumor recurrence (17). Bojoga et al. (18) have explored the relationship between systemic inflammation and thyroid cancer by focusing on the inflammatory mediator PTX3. While PTX3 plasma levels were not significantly different between overall thyroid cancer cases and benign thyroid disease, markedly elevated levels and strong tissue expression were observed in aggressive subtypes, particularly anaplastic thyroid cancer. These findings suggest a potential association between PTX3 and tumor aggressiveness, supporting the need for further investigation into its biological role and possible value as a biomarker in advanced thyroid cancer.

Here we assess plasmatic PTX3 levels both preoperatively and postoperatively in patients who have undergone hemi- or total thyroidectomy for benign or malignant nodules. Additionally, we investigate the pattern of PTX3 expression in thyroid carcinoma samples and examine its association with different immune cell types.

## 2 Materials and methods

### 2.1 Ethics approval and patients

This prospective monocentric study was conducted at Humanitas Mater Domini Clinical Institute (Castellanza, Italy) in accordance with the ethical principles outlined by the Declaration of Helsinki and was approved by the Institutional Review Board of Humanitas Research Hospital (approval HDM 285/19). Patients with nodular thyroid disease, defined as the presence of one or more thyroid nodules requiring surgical intervention, were prospectively enrolled in this study. Surgical indications were established according to international guidelines and included: a) suspicious or malignant cytology on fine-needle aspiration (FNA), typically corresponding to Bethesda categories IV, V, or VI; b) large nodules (>3–4 cm) associated with compressive symptoms, such as dysphagia or dyspnea; c) documented progressive nodular enlargement; or d) concomitant hyperthyroidism. Although the cohort included patients with autoimmune thyroiditis and hyperfunctioning nodules or toxic goiter, no cases of Graves’ disease were identified. The study population therefore encompassed a broad spectrum of thyroid disorders, ranging from benign multinodular goiter to differentiated thyroid carcinoma. Eligible participants were scheduled to undergo either total thyroidectomy or hemithyroidectomy. All participants gave written informed consent. The exclusions criteria of the study were: age under 18 years old and no acquisition of the informed consent. Moreover, patients with the following conditions were not represented in the dataset: a) active acute systemic infections; b) active inflammatory or autoimmune diseases (e.g., systemic lupus erythematosus); and c) active malignancies undergoing treatment. Among the cohort, the seven patients with a prior history of cancer were all in documented long-term complete remission and no longer receiving therapy. The seven patients with a history of autoimmune disease included five cases of stable chronic autoimmune thyroiditis (Hashimoto’s thyroiditis), one case of celiac disease, and one case of rheumatoid arthritis in sustained clinical remission. All patients underwent surgical operation at Humanitas Mater Domini Clinical Institute between May 2019 and January 2022. To ensure the validity of the 45-day postoperative assessment, patients were prospectively monitored for intercurrent events. No patients in the cohort developed acute infections, required initiation of anti-inflammatory or immunosuppressive therapies, or underwent additional surgical procedures during the 45-day follow-up period. Both blood and tissue samples were obtained from the same cohort of prospectively recruited surgical patients. Eighteen healthy volunteers were enrolled as controls. Individuals with a history of thyroid disease, autoimmune disorders, acute or chronic inflammatory conditions, malignancy, or ongoing treatment potentially affecting inflammatory biomarkers were excluded from participation.

### 2.2 Blood samples

To evaluate plasmatic PTX3 levels, antecubital venous blood sample was taken right before the surgical procedure, and a second venous blood sample was taken 45 days after surgery during routine checkup for hormone replacement therapy. Blood samples were centrifugated at 4000 RPM for 10 minutes. Plasma was separated, stored in 1.5 ml Eppendorf tubes and frozen at -20 °C in a controlled freezer. An identification code label was placed on each tube to ensure anonymity. The material was then sent at -20 °C to the Immunopharmacology Laboratory of IRCCS Humanitas Research Hospital, Rozzano, Milan, Italy. PTX3 levels were evaluated by ELISA assay as previously described (19). All analyses were conducted without access to the patients’ clinical information.

### 2.3 Tissue microarray

Thyroid tissue samples were analyzed using a homemade tissue microarray (TMA). The TMA paraffin block was composed by cylindrical cores deriving from formalin fixed paraffin embedded (FFPE) tissue of different patients retrieved from the archive of Pathology, IRCCS Humanitas research Hospital. A sampling needle (1 mm) punches the core from the donor block and places it in the predesigned recipient block. An average of three cores per patient were placed on different spots of the TMA recipient block. The tissue cores derived from patients with neoplastic disease as well as inflammatory disease. The cohort comprised healthy tissues (n = 21), thyroiditis (n = 49), thyroid follicular nodular disease (n = 88), thyroid adenomas (n = 9) and thyroid cancers (n = 41). The malignant group included 14 tissue cores from patients with papillary thyroid carcinoma (11 classic variant and 3 follicular variant papillary thyroid carcinoma), 2 cores from follicular carcinoma, and 1 core from medullary carcinoma. Histopathological diagnoses were reviewed by an experienced endocrine pathologist according to the current WHO classification of thyroid tumors. In cases of follicular variant papillary thyroid carcinoma, all lesions were re-evaluated for the presence of capsular and/or vascular invasion. Tumors lacking evidence of invasion and fulfilling the diagnostic criteria for non-invasive follicular thyroid neoplasm with papillary-like nuclear features (NIFTP) were excluded from the study cohort.

### 2.4 Histochemistry

TMAs were sectioned to a thickness of 3 µm and then immersed in a fresh Hematoxylin and Eosin solution for general staining.

### 2.5 Immunohistochemistry

3 µm-thick TMAs sections were immersed in a heat retrieval solution (DIVA Antigen Decloaker solution, Biocare Medical, USA) for antigen unmasking using a pressure cooker (Decloaking Chamber™, Biocare Medical, USA). Tissue slides were treated with 3% hydrogen peroxide solution for 10 minutes at room temperature (RT) to quench endogenous peroxidase. Tissue slides were then blocked with Background Sniper (Biocare Medical, USA) for 20 min at RT and incubated with antibodies raised against CD68 (Dako, clone KP1, dilution 1:100), Tryptase (NeoBiotechnology, clone TPSAB1/1963, dilution 1:200) or PTX3 [Homemade, Rabbit Polyclonal antibody, 0.25 µg/mL, dilution 1: 4000, (9, 20, 21)] for 1 hour at RT. Tissue sections were incubated with the MACH1 mouse probe (Biocare Medical, USA) for 15 minutes at RT, followed by MACH1 HRP-polymer for another 15 minutes at RT. For rabbit antibodies, sections were incubated exclusively with MACH1 HRP-polymer (Biocare Medical, USA) for 30 minutes at RT. The chromogen reaction was developed using Betazoid DAB Chromogen Kit (Biocare Medical) and the sections were counterstained with hematoxylin. Whole tissue slides were acquired at 20x (Objective magnification) using the automatic slide scanner Zeiss Axioscan Z1 (Zeiss, Italy). Immunoreactivity was assessed semi-quantitatively by an expert pathologist (MC).

### 2.6 Statistical analysis

Statistical analyses were performed using the Statistical Package for Social Sciences (SPSS, IBM Corp., Armonk, NY, USA) and GraphPad Prism 11 (GraphPad Software, San Diego, CA, USA) for descriptive statistics. Normality of continuous variables was assessed using the Shapiro–Wilk test. Based on distribution, univariate comparisons were conducted using Student’s t-test for normally distributed data or the Mann–Whitney U test for non-parametric data. Pearson correlation coefficients were used to assess collinearity between variables. Postoperative PTX3 changes were evaluated by calculating ΔPTX3 as the individual difference between postoperative and preoperative values, and paired comparisons were performed using the Wilcoxon signed-rank test. This approach allowed assessment of within-patient variability and postoperative dynamics. Statistical significance was set at p < 0.05.

## 3 Results

### 3.1 Patient characteristics and PTX3 plasma levels

**Figure 1:**
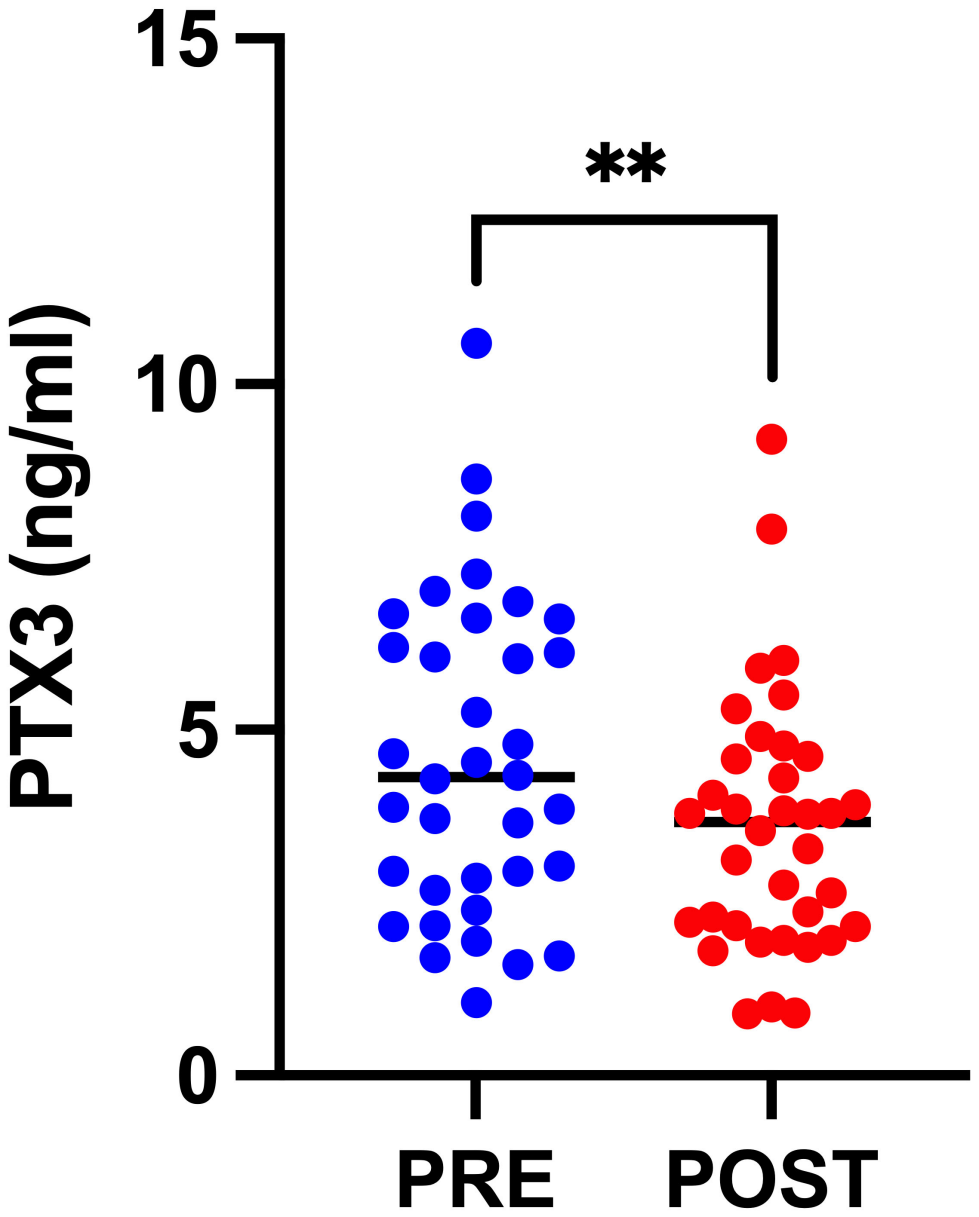
Preoperative and postoperative PTX3 plasma levels in 36 matched subjects, regardless of thyroid disease. The asterisk (**) denotes statistical significance (P < 0.01).

A total of 58 patients were initially enrolled in the study; among them 6 were excluded because preoperative PTX3 blood levels were unavailable, resulting in 52 patients included in the final analysis (**Table 1**). Of these, 44 (84.6%) were female, with a mean age of 56 years (range: 25–79 years). Seven patients (13.5%) had a prior history of cancer, and 7 (13.5%) had an autoimmune disease. Preoperative subclinical hyperthyroidism was observed in 2 cases (3.8%). Forty-three patients (82.7%) underwent total thyroidectomy, whereas nine patients (17.3%) received hemithyroidectomy. Histopathological examination revealed malignant tumors in 21 patients (40.4%), with 18 cases (34.6%) diagnosed as papillary carcinoma, 2 cases (3.8%) as follicular carcinoma, and 1 case (1.9%) as oncocytic cell carcinoma. The mean preoperative CRP level was 0.37 ± 0.42 mg/L (**Table 1**). Preoperative PTX3 levels averaged 4.58 ± 2.29 ng/mL and were significantly higher than those measured in 18 healthy volunteers (mean age 57 ± 1 years), in whom the average level was 2.12 ± 0.91 ng/mL. Compared with preoperative levels, postoperative PTX3 concentrations decreased significantly to a mean of 3.58 ± 1.86 ng/mL (p = 0.006) (**Figure 1**). Although PTX3 levels tended to be higher in malignant nodules, no statistically significant difference was observed between patients with benign versus malignant disease (4.57 ± 2.54 ng/mL vs. 4.61 ± 1.85 ng/mL). No significant correlations were found between elevated PTX3 levels (defined as >2 ng/mL) and other variables, including nodule size, autoimmune disease, prior cancer history, or mean preoperative CRP (**Table 2**). In patients with paired pre- and postoperative plasma samples (n = 36), stratified analyses were performed in benign (n = 23) and malignant (n = 13) subgroups. As shown in **Table 3**, a postoperative decrease in PTX3 levels was observed consistently in both subgroups. The stronger statistical significance (p = 0.006) observed in the pooled cohort compared with subgroup analyses is most plausibly explained by the increased statistical power associated with the larger sample size, while the direction of the observed changes remained concordant across both benign and malignant groups. In patients with available postoperative samples (n = 40), PTX3 levels were compared between patients undergoing total thyroidectomy (n = 32) and those undergoing hemithyroidectomy (n = 8). No statistically significant difference was found between the two groups (3.44 ± 1.76 ng/mL vs. 3.68 ± 2.30 ng/mL, respectively; p = 0.877).

**Table 1:** Epidemiological, clinical, and histopathological characteristics of the subjects included in the study.

Variable	Value in Patients (n = 52)
Age (years)	56.0 ± 14.6
Sex (female, %)	44 (84.62%)
Autoimmune diseases	7 (13.46%)
Previous cancer history	7 (13.46%)
Hyperthyroidism (TSH<0.34)	2 (3.85%)
C-Reactive Protein (mg/L)	0.37 ± 0.41
Surgery (Hemithyroidectomy)	9 (17.31%)
Thyroiditis	12 (23.08%)
Hyperplasia	26 (50.00%)
Adenoma	5 (9.62%)
Malignant disease	21 (40.38%)
Papillary carcinoma	18 (34.62%)
Follicular carcinoma	2 (3.85%)
Oxhyphilic cell carcinoma	1 (1.92%)

Data are presented as mean ± standard deviation (SD) for normally distributed variable and as number and frequency for categorical variables.

**Table 2:** Baseline characteristics of patients stratified by high and low plasmatic levels of PTX3.

Variable	PTX3 ≤ 2 ng/mL	PTX3 >2 ng/mL	p
	n = 8	n = 44	
Age (years)	50.1 ± 6.9	57.0 ± 15.4	0.221
Sex (female, n, %)	6 (75.00%)	38 (86.36%)	0.593
Autoimmune diseases (n, %)	2 (25.00%)	5 (11.36%)	0.291
Previous cancer (n, %)	1 (12.50%)	6 (13.64%)	0.930
C-reactive protein (ng/ml)	0.21 ± 0.13	0.42 ± 0.46	0.504
Thyroiditis (n, %)	2 (25.00%)	10 (22.73%)	0.888
Malignant (n, %)	2 (25.00%)	19 (43.18%)	0.449

Data are presented as mean ± standard deviation (SD) for normally distributed variables for non-normally distributed variables, and as number and percentage for categorical variables. Comparisons between groups were performed using Student’s t-test for normally distributed variables, the Mann–Whitney U test for non-normally distributed variables, and the chi-square test for categorical variables.

**Table 3:** Pre- and postoperative plasma PTX3 levels in patients with benign and malignant disease.

Patient group	PTX3a (ng/mL)	PTX3b (ng/mL)	Δ PTX3	p
All (n=36)	4.34 (2.39–6.19)	3.55 (2.17–4.90)	- 1.00 ng/mL	0.006
Benign (n=23)	3.88 (2.52–6.40)	3.28 (1.96–4.77)	- 1.04 ng/mL	0.058
Malignant (n=13)	4.34 (3.03–6.19)	3.83 (2.22–4.90)	- 0.95 ng/mL	0.048

Data are presented as median (IQR). PTX3a and PTX3b denote preoperative and postoperative plasma PTX3 levels, respectively. Pre- and postoperative comparisons were performed using paired analyses with the Wilcoxon signed-rank test.

### 3.2 PTX3 immunoreactivity assessment in tissue samples

**Figure 2:**
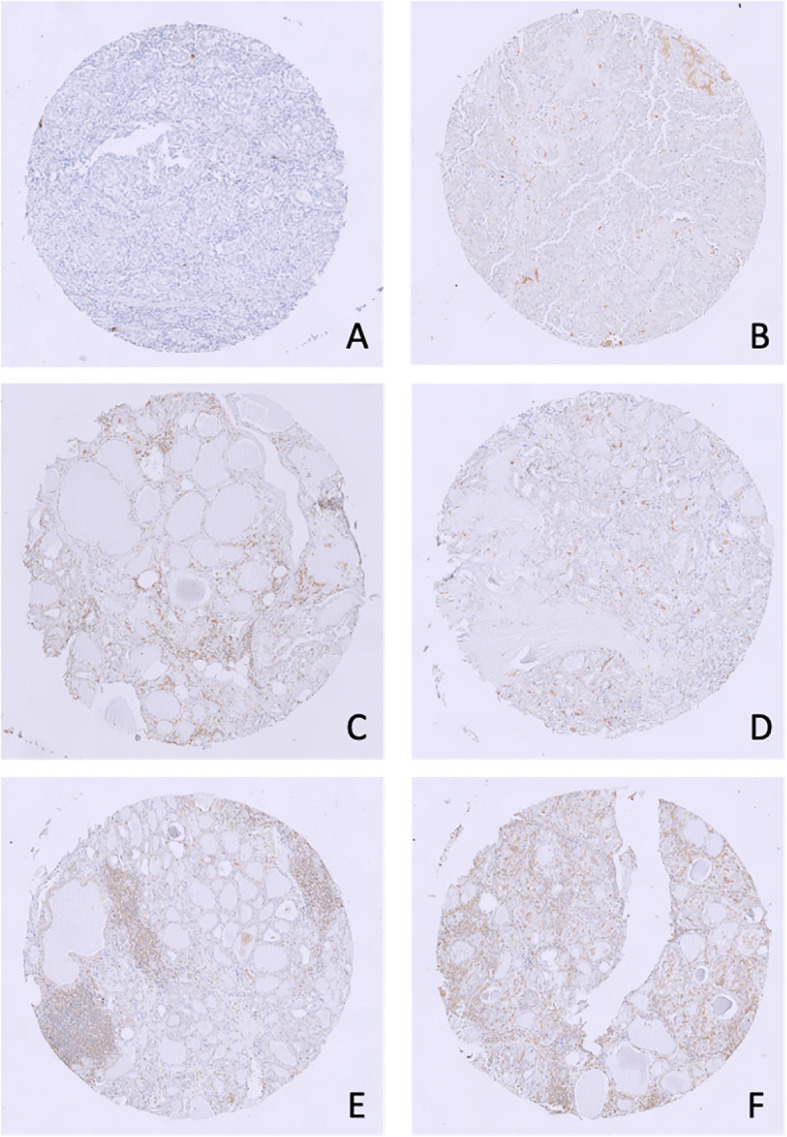
PTX3 expression: papillary carcinoma showed weak **(A)** or absent **(B)** immunoreactivity. Focal PTX3 staining was observed in thyroiditis **(C)** and in the follicular variant of papillary carcinoma **(D)** from the same patient. Strong, diffuse positivity was evident in two distinct cases of thyroiditis **(E, F)**.

**Figure 3:**
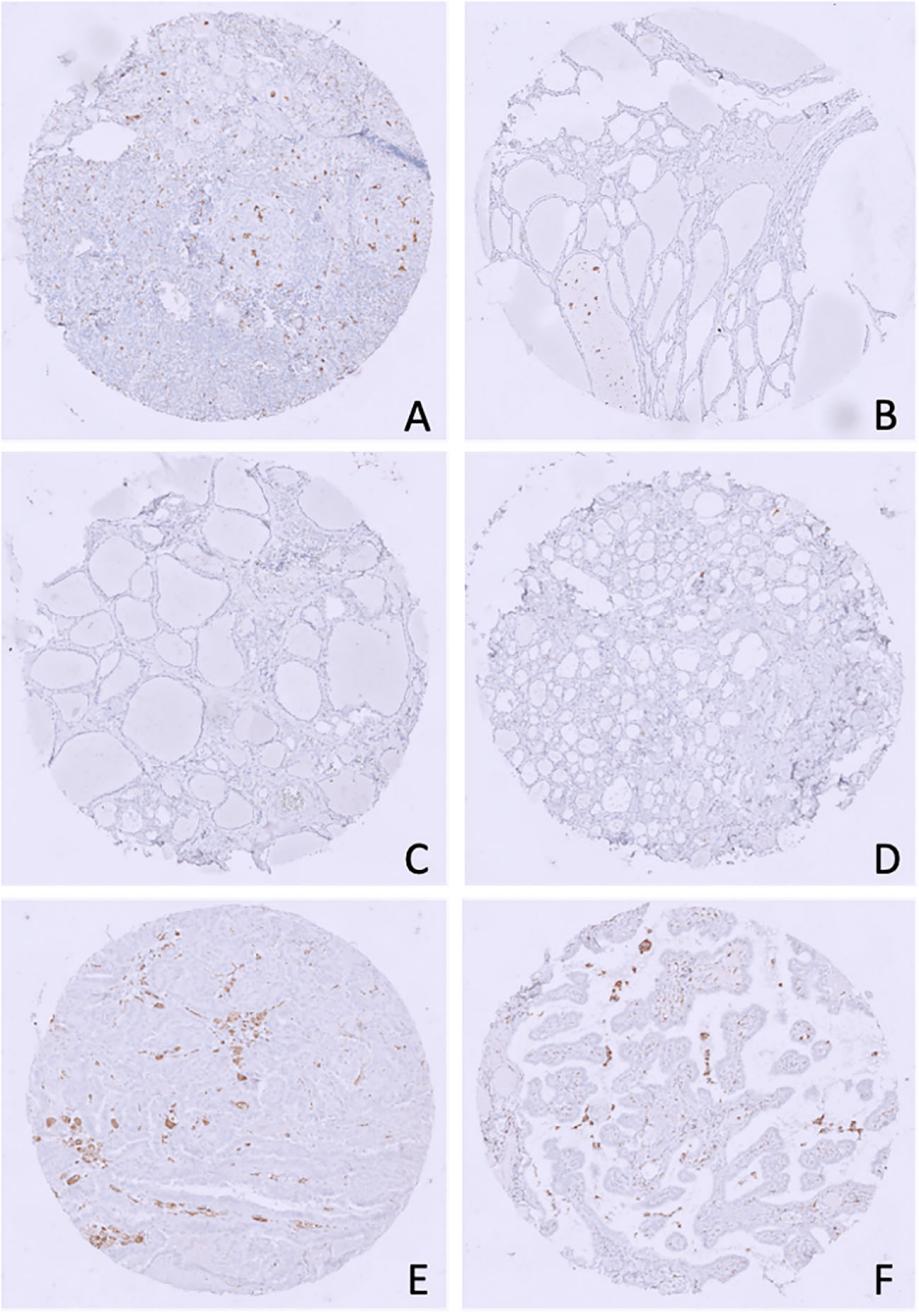
CD68 expression: CD68^+^ macrophages were predominantly absent or scarce in normal tissue from patients with carcinoma **(A)** and thyroiditis **(B)**. In thyroiditis, most samples were negative or showed low positivity **(C, D)**. In carcinoma tissues, some cases exhibited moderate macrophage infiltration **(E, F)**.

**Figure 4:**
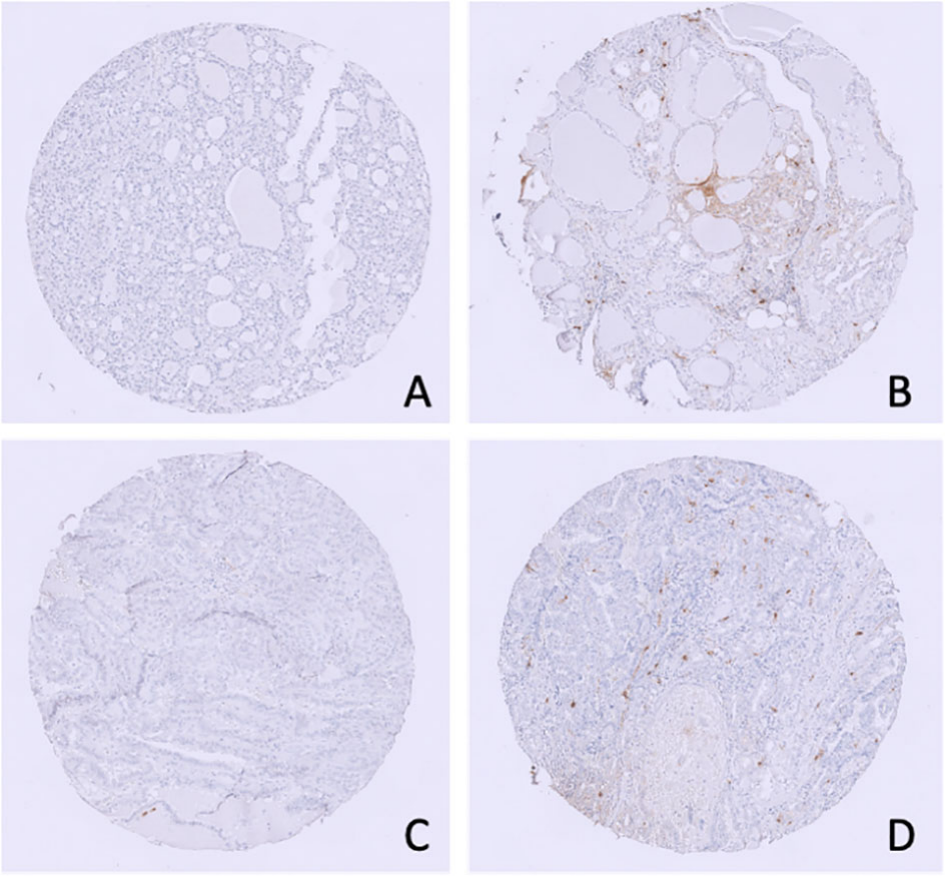
Tryptase expression: most thyroiditis samples were negative **(A)** or showed minimal positivity **(B)**, as observed also in carcinoma tissues **(C, D)**.

The expression of PTX3, CD68, and tryptase differed across the analyzed tissue groups (**Figures 2**–**4**; **Supplementary Table 1**). In healthy tissues (n = 21), PTX3 expression was absent in all cases (100% negative). CD68 was predominantly negative (66.7%), with limited positivity (19%) while tryptase expression was largely negative (85.7%), with minimal positivity (9.5%). In thyroiditis (n = 49), PTX3 showed a marked increase in positivity (81.6%), whereas CD68 displayed a relatively balanced distribution between positive (47%) and negative (43%) cases. Tryptase remained mostly negative (79.6%). In thyroid follicular nodular disease (n = 88), PTX3 positivity was observed in 58% of cases, while CD68 was more frequently negative (52.3%) than positive (36.4%), and tryptase was predominantly negative (81.9%). In adenomas (n = 9), PTX3 was positive in 66.7% of cases, CD68 expression was mainly positive (66.7%), and tryptase showed moderate positivity (55.6%). In malignant tissues (n = 41), PTX3 expression was strongly positive in most cases (87.8%), with very few negative samples. CD68 also showed higher positivity (51.2%) compared to negativity (36.6%), while tryptase exhibited a more balanced distribution between positive (41.4%) and negative (48.8%) cases. Notably, both mast cells and macrophages were more abundant in carcinoma tissues, irrespective of histotype, compared to normal parenchyma (**Figures 3**, **4**). Furthermore, a statistically significant association was found between mast cell presence and thyroid carcinoma (p = 0.0025).

## 4 Discussion

The present study demonstrated that preoperative PTX3 plasma levels were significantly elevated in patients with thyroid disease compared with healthy controls and decreased significantly following surgery. This postoperative reduction was consistent across both benign and malignant subgroups and was independent of the extent of thyroid resection and other clinical variables. In parallel, tissue analysis revealed progressively increased PTX3 immunoreactivity from healthy thyroid tissue to inflammatory, benign, and malignant lesions, with a significant association between mast cell presence and thyroid carcinoma. These findings suggest that PTX3 may reflect an underlying inflammatory microenvironment associated with thyroid disease rather than being specific to malignancy. The consistent postoperative decline further indicates that thyroidectomy may reduce a systemic or local source of PTX3, supporting a potential link between thyroid tissue–associated inflammation and circulating PTX3 levels. Recently, Bojoga et al. (18) assessed PTX3 plasma levels and tissue expression in non-medullary thyroid cancer. They found that PTX3 levels were similar to those measured in controls but were significantly higher in poorly differentiated and anaplastic thyroid cancers. Strong PTX3 tissue expression was seen in most anaplastic thyroid cancer cases, while benign and differentiated tissues showed minimal staining. Anaplastic thyroid cancer samples were CD68^+^, suggesting macrophage infiltration, with few cells co-expressing PTX3 and CD68. These results indicate a potential link between PTX3 and aggressive thyroid cancer, especially anaplastic thyroid cancer, warranting further investigation. In our current study we investigated the role of PTX3 in patients with thyroid nodular disease, focusing on both systemic plasma levels and local tissue expression. Our findings offer novel insights into the potential involvement of PTX3 in the inflammatory and remodeling processes associated with thyroid pathology.

We found that the mean preoperative plasma PTX3 level in patients undergoing thyroidectomy for nodular disease was significantly higher than that observed in a group of healthy subjects. This elevated systemic level suggests that the presence of thyroid nodules, irrespective of their benign or malignant nature, may be associated with underlying systemic or local inflammation that stimulates PTX3 production. Interestingly, although PTX3 levels tended to be higher in malignant nodules, no statistically significant difference was observed between patients with benign and malignant disease. The consistency in the direction of postoperative PTX3 changes across benign and malignant subgroups supports the interpretation that these findings reflect a shared underlying biological response rather than subgroup-specific effects or methodological differences, with the greater statistical significance observed in the pooled analysis primarily driven by increased statistical power (**Table 3**). This finding suggests that elevated circulating PTX3 reflects a general response to the presence of thyroid nodules and the associated inflammatory microenvironment, rather than being specifically driven by tumorigenesis. Consequently, while PTX3 may represent a sensitive marker of thyroid pathology, its utility as a standalone diagnostic biomarker for malignancy appears limited. The statistically significant reduction in plasma PTX3 levels postoperatively supports the idea that the thyroid pathology itself is a primary source or trigger for the elevated plasmatic PTX3. A potential concern arising from the inclusion of both total and partial thyroidectomies is that residual thyroid tissue could continue to secrete PTX3, potentially confounding postoperative findings. Interestingly, our subgroup analysis showed no significant difference in postoperative PTX3 levels between patients undergoing total thyroidectomy and those undergoing hemithyroidectomy (p = 0.877). This finding suggests that healthy, non-pathological thyroid tissue is not a major source of circulating PTX3. Instead, it supports the hypothesis that preoperative PTX3 elevation is primarily associated with localized stress within nodular tissue, which is removed during surgery.

The removal of the affected thyroid tissue seems to reduce the stimulus that leads to PTX3 production and release. This observation reinforces the link between the pathological thyroid tissue and systemic PTX3 elevation. Furthermore, the lack of correlation between elevated PTX3 and nodule dimension, autoimmune diseases, previous cancer, or CRP further highlights PTX3 as an independent marker specifically related to the nodular/surgical pathology.

Immunohistochemistry analysis provided critical context for the systemic findings by examining PTX3 expression patterns within the thyroid tissue. We observed a statistically significant correlation between PTX3 expression and thyroiditis (p=0.001), with moderate to diffuse positivity in 81.6% of thyroiditis cases. This result aligns with PTX3’s known role as an acute-phase inflammatory mediator. Thyroiditis, an inflammatory condition of the thyroid, appears to be a robust local inducer of PTX3 expression. The lower or negative expression in healthy tissue, thyroid adenoma, and most follicular nodular disease further supports its role as a marker of active inflammation or tissue remodeling. Regarding tissue characterization, healthy thyroid tissues consistently exhibited complete absence of PTX3 immunoreactivity. In contrast, a low but detectable level of PTX3 expression was observed in most malignant samples. Although both staining intensity and the proportion of positive cells remained overall low, this represents a significant relative increase compared with the entirely negative baseline observed in healthy tissues (p < 0.05). This moderate expression was noted in 19% of malignant cases, all of which were Follicular Variant of Papillary Carcinoma (FVPTC) or a combination of classical and follicular variant. This finding is particularly relevant when considered alongside the established role of PTX3 as an “extrinsic oncosuppressor” in other cancers (13, 22–24). Low expression of PTX3 in the majority of malignant samples also aligns with the findings of Luo et al. (17), who identified PTX3 as part of a four-gene signature (PAPSS2, PCOLCE2, PTX3, and TGFBR3) for determining the prognosis of papillary thyroid carcinoma. In their bioinformatics analysis, a low-risk score, associated with a significantly more favorable overall survival (OS), was dependent on the expression pattern of these four genes. Importantly, their findings indicated that PTX3 expression was significantly lower in patients surviving with tumors than in tumor-free patients, revealing a potential correlation between PTX3 and tumor recurrence (17). This suggests that a loss of PTX3 expression in papillary thyroid carcinoma may reflect a more aggressive tumor phenotype or an evasion of the innate immune response that PTX3 is meant to regulate.

The relatively low absolute tissue expression of PTX3 observed in most thyroid carcinoma samples warrants careful interpretation within the broader context of cancer biology. PTX3 is increasingly recognized as having a dual, context-dependent role in malignancy. On one hand, our hypothesis that reduced PTX3 expression may be associated with an adverse prognostic profile is supported by evidence identifying PTX3 as an extrinsic oncosuppressor. Seminal work by Bonavita et al. (9) demonstrated that PTX3 can inhibit tumor progression by modulating complement activation and limiting macrophage-driven pro-tumorigenic inflammation. In this framework, the low expression detected in differentiated thyroid carcinomas may reflect a partial loss of this protective, oncosuppressive component of the tumor microenvironment, potentially contributing to immune evasion or tumor progression. On the other hand, PTX3 function appears highly context-dependent, with opposite associations reported in other tumor types. For example, increased PTX3 expression has been linked to poor prognosis in B-cell lymphoma (25). This variability suggests that the biological role of PTX3 is shaped by the specific cytokine milieu and cellular composition of each tumor microenvironment. Accordingly, while our preliminary findings in thyroid nodular disease demonstrate a significant relative increase in PTX3 compared with a completely negative healthy baseline, the low absolute expression observed in carcinomas highlights the need for further functional studies. These are required to clarify whether PTX3 primarily reflects a protective inflammatory response or represents a secondary, context-dependent bystander in thyroid tumorigenesis. Furthermore, the moderate PTX3 expression observed specifically in the follicular variant of papillary thyroid carcinoma (FVPTC) subset is intriguing. As highlighted by Daniels et al. (26), FVPTC is a heterogeneous entity, with different subsets [e.g., noninvasive follicular thyroid neoplasm with papillary-like nuclear features (NIFTP) versus invasive FVPTC) exhibiting distinct clinical behaviors and molecular profiles (RAS vs. BRAF mutations). The presence of moderate PTX3 expression in some FVPTC cases may correlate with the inflammatory or tissue remodeling activity prevalent in the more invasive or aggressive subsets of FVPTC, or perhaps a localized, TSH-driven inflammatory reaction in the tumor microenvironment (12). A detailed molecular and clinical sub-classification of our FVPTC cohort will be necessary to determine if PTX3 expression aligns with a specific molecular or clinical behavior.

Lastly, we found a significant correlation (p=0.0025) between the presence of mast cells and carcinoma tissue compared to normal parenchyma. CD68^+^ macrophages were likewise observed at higher abundance within carcinoma tissue. The co-localization and association of the inflammatory marker PTX3 in thyroiditis with key immune cells (namely mast cells and macrophages in carcinoma) highlight the pivotal interplay among inflammation, innate immune responses, and thyroid pathology.

This study has several limitations, including its single-center design and relatively small sample size. While the statistically significant findings are compelling, a larger, multicentric cohort is needed to validate the plasma PTX3 cutoff and its potential clinical role. Additionally, the study used a broad classification (benign vs. malignant) for plasma PTX3 analysis; future studies should differentiate specific benign and malignant histotypes to better understand the nuances of PTX3 involvement. The tissue analysis of FVPTC must be refined to distinguish between NIFTP and the truly invasive variants to precisely correlate PTX3 expression with the known molecular and prognostic categories of this complex disease. A further limitation of this study is the absence of rare thyroid carcinoma subtypes, including undifferentiated and medullary thyroid carcinomas. Therefore, the applicability of our findings to these tumor entities remains to be established. Future studies should aim to: validate the prognostic significance of PTX3 expression in PTC in a larger cohort and correlate it with survival outcomes, in line with the findings of Luo et al. (17); more precisely identify the cellular sources of PTX3 across different thyroid pathologies (e.g., follicular cells, inflammatory cells, fibroblasts); and examine PTX3 expression in FVPTC in relation to the NIFTP/invasive classification and key molecular markers (such as BRAF and RAS mutations) to assess whether PTX3 indicates the more aggressive, cPTC- or FTC-like subtypes.

This study is the first to assess both systemic and local PTX3 in thyroid nodular disease. Elevated preoperative plasma PTX3 levels that decrease significantly after surgery indicate that PTX3 reflects active thyroid pathology or stress, although it does not specifically discriminate malignancy. Tissue analyses showing high PTX3 in thyroiditis, low expression in most carcinomas, which may be linked to poor prognosis, and distinct patterns in the heterogeneous FVPTC subset suggest that PTX3 could play a role in innate immune and inflammatory responses underlying thyroid remodeling and disease. In conclusion, these results highlight the involvement of PTX3 in thyroid-associated inflammation; however, further studies are needed to determine whether this molecule may have prognostic value or contribute to clinical decision-making. Accordingly, these findings should be regarded as exploratory and require validation in larger prospective cohorts.

## Data Availability

The original contributions presented in the study are included in the article/**Supplementary Material**. Further inquiries can be directed to the corresponding author.
