# Supplementary material for: Pentraxin-3 in thyroid nodules: a systemic marker of pathology and a local mediator of inflammation and carcinoma
Source: Front Endocrinol (Lausanne). 2026 Jul 17;17:1847072. doi: 10.3389/fendo.2026.1847072 (PMC13423634; doi:10.3389/fendo.2026.1847072)
Supplement: Supplementary file 1 [file Table1.docx]

**Table S1**. Expression of PTX3, CD68, and tryptase in the analysed tissues.

|  |  |  | **PTX3** | | |  | **CD68** | | |  | **Tryptase** | | |
| --- | --- | --- | --- | --- | --- | --- | --- | --- | --- | --- | --- | --- | --- |
|  | n |  | POS | NEG | NE |  | POS | NEG | NE |  | POS | NEG | NE |
| **Healthy** | 21 |  |  | 21  (100 %) |  |  | 4  (19%) | 14  (66.7%) | 3  (14.3%) |  | 2  (9.5%) | 18  (85.7%) | 1  (4.8%) |
|  |  |  |  |  |  |  |  |  |  |  |  |  |  |
| **Thyroiditis** | 49 |  | 40  (81.6%) | 6  (12.3%) | 3  (6.1%) |  | 23  (47%) | 21  (43%) | 5  (10%) |  | 9  (18.4%) | 39  (79.6%) | 1  (2%) |
|  |  |  |  |  |  |  |  |  |  |  |  |  |  |
| **Thyroid follicular nodular disease** | 88 |  | 51  (58%) | 26  (29.5%) | 11  (12.5%) |  | 32  (36.4%) | 46  (52.3%) | 10  (11.3%) |  | 6  (6.8%) | 72  (81.9%) | 10  (11.3%) |
| **Adenoma** | 9 |  | 6  (66.7%) | 3  (33.3%) | 0 |  | 6  (66.7%) | 1  (11.1%) | 2  (22.2%) |  | 5  (55.6%) | 3  (33.3%) | 1  (11.1%) |
| **Malignant** | 41 |  | 36  (87.8%) | 2  (4.9%) | 3  (7.3%) |  | 21  (51.2%) | 15  (36.6%) | 5  (12.1%) |  | 17  (41.4%) | 20  (48.8%) | 4  (9.8%) |
|  |  |  |  |  |  |  |  |  |  |  |  |  |  |

**Notes:** Data are expressed as n (%). POS: Immunoreactivity detected. NEG: No staining observed. NE: Not evaluable due to partial or complete core detachment.
